# Whole-Body Perfusion in Neonates and Infants Undergoing Aortic Arch Surgery—Working Towards the New Standard

**DOI:** 10.3390/jcm13206170

**Published:** 2024-10-16

**Authors:** Rodrigo Sandoval Boburg, Isabelle Doll, Christian Jörg Rustenbach, Rafal Berger, Walter Jost, Harry Magunia, Johannes Nordmeyer, Jörg Michel, Christian Schlensak

**Affiliations:** 1 Department of Thoracic and Cardiovascular Surgery, University Hospital Tübingen, Eberhard-Karls-University Tübingen, Hoppe-Seyler-Str. 3, 72076 Tübingen, Germanychristian.rustenbach@med.uni-tuebingen.de (C.J.R.); walter.jost@med.uni-tuebingen.de (W.J.);; 2 Department of Anaesthesiology and Intensive Care Medicine, University Hospital Tübingen, Eberhard-Karls-University Tübingen, Hoppe-Seyler-Str. 3, 72076 Tübingen, Germany; 3 Department of Paediatric Cardiology and Intensive Medicine, University Hospital Tübingen, Eberhard-Karls-University Tübingen, Hoppe-Seyler-Str. 3, 72076 Tübingen, Germany

**Keywords:** whole-body perfusion, neonatal aortic arch surgery, selective cerebral perfusion

## Abstract

**Background:** Neonatal aortic arch surgery remains one of the most challenging procedures in congenital cardiac surgery. In recent years, there has been a trend away from selective cerebral perfusion (SCP) and arrest of body circulation towards whole-body perfusion (WBP), a combination of SCP and lower-body perfusion (LBP), to facilitate arch surgery and preserve organ function. **Methods:** Retrospective, single-centre analysis was conducted of patients under one year of age undergoing aortic arch surgery from January 2014 until December 2022. SCP was used from January 2014—December 2017; WBP was implemented from January 2017—December 2022. Patients were separated according to the type of perfusion used during surgery, SCP or WBP. The cohort consisted of a total of 95 patients, 34 in the SCP group and 61 in the WBP group. **Results:** Patients in the WBP group showed significantly lower rates of intraoperative transfusions, namely red blood cells, fresh-frozen plasma and thrombocytes (*p*-value < 0.01, <0.01, and <0.01, respectively). The WBP group showed significantly lower creatinine and higher urine output values 24 and 72 h after surgery (*p*-value = 0.02, <0.01, respectively). The WBP group showed a significant lower incidence of major neurological complications (*p*-value 0.01). Binary logistic regression analyses showed favourable outcomes for the WBP group regarding 30-day mortality (OR = 0.03, CI = 0.003–0.391, *p*-value = <0.01), multiorgan failure (OR = 0.002, CI = 0–0.275, *p*-value = 0.01), neurological complications (OR = 0.994, CI = 0.998–1, *p*-value = 0.06) and postoperative renal replacement therapy (RRT) (OR = 0.989, CI = 0.983–0.995, *p*-value = <0.01). **Conclusions:** Patients with WBP received fewer intraoperative transfusions, showed improved postoperative renal function and suffered significantly fewer neurological complications.

## 1. Introduction

Neonatal aortic arch surgery poses a significant challenge to the managing team. One of the primary difficulties is achieving an adequate perfusion during cardiopulmonary bypass (CPB). Perfusion techniques have evolved from deep hypothermic circulatory arrest (DHCA) to selective cerebral perfusion (SCP) and, in recent years, there has been a growing trend towards a combination of SCP and lower-body perfusion (LBP): the so-called whole-body perfusion (WBP) [1,2,3,4,5,6,7].

LBP can be achieved through a direct cannulation of the descending aorta. Different techniques have been described by several groups. It can be achieved through a left-sided thoracotomy or by making an incision in the posterior pericardium [6,8,9,10]. Our group previously described a technique in which LBP was achieved by placing an arterial sheath in the common femoral artery during anaesthesia induction [7].

Our initial analyses were able to determine that an adequate LBP led to improvement of intraoperative transfusions and postoperative complications, especially neurological ones, compared to patients who underwent the same type of surgery using an SCP strategy [7,11,12]. Due to the small number of patients undergoing this type of surgery, our initial analyses were performed with small cohorts.

The aim of this study is to compare major postoperative complications between patients who underwent aortic arch reconstruction surgery using SCP and WBP 6 years after the implementation of the WBP technique in our centre. This comparison will provide valuable insights into the efficacy and safety of WBP in neonatal aortic arch surgery.

## 2. Methods

### 2.1. Patient Selection and Grouping

**Figure 1:**
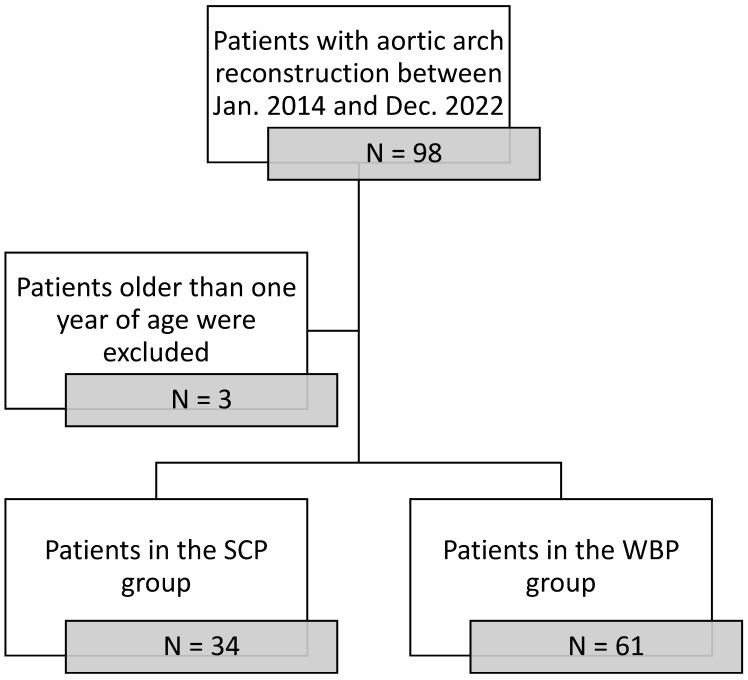
Patient-selection process.

We retrospectively screened all paediatric patients under one year of age who underwent aortic arch surgery at the University Hospital Tübingen from January 2014 to December 2022. Patients were eligible for this study if they underwent surgeries such as the Norwood procedure, Damus–Kaye–Stansel anastomoses or an aortic arch reconstruction due to hypoplasia, either as a primary or a redo surgery. Figure 1 shows a patient selection chart. Before January 2017, all patients undergoing any of these procedures were treated with SCP and moderate hypothermia (26–28 °C). After January 2017, a WBP technique was implemented in our centre consisting of the standard SCP with LBP and mild hypothermia (30–32 °C). All paediatric patients undergoing aortic arch surgery after this date were treated with WBP. After selection, there were 61 patients in the WBP group and 34 patients in the SCP group. All patients were treated by the same experienced surgeon, perfusionist and the same team of experienced cardiac anaesthesiologists throughout the treatment period.

### 2.2. Perfusion Strategies

SCP was established through a polytetrafluoroethylene (PTFE) shunt anastomosed to the brachiocephalic trunk where the arterial cannula was inserted. During SCP, perfusion was established at a rate of 50–80 mL/kg and monitored with bi-frontal near-infrared spectroscopy. Patients in this group were cooled down to 26–28 °C during aortic arch repair; after the repair was finished and complete CPB was reinstated, patients were warmed up.

**Figure 2:**
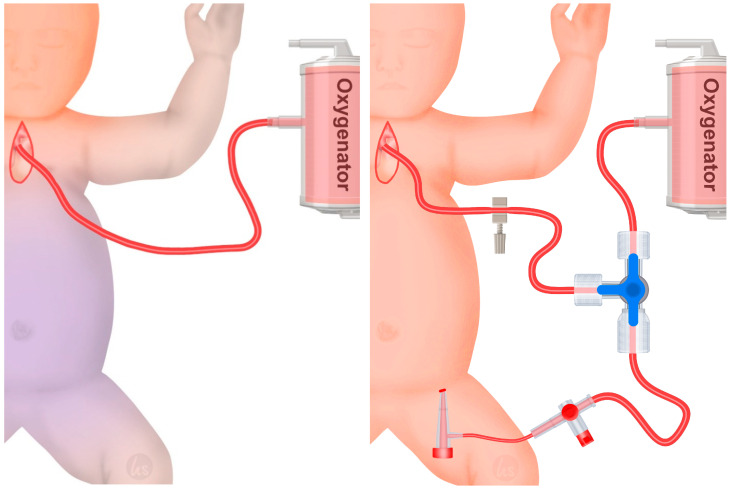
**Left:** Selective cerebral perfusion (SCP) is achieved by cannulating the brachiocephalic trunk. **Right:** Whole-body perfusion (WBP) is achieved by establishing SCP and cannulating the common femoral artery with a 4 Fr. arterial sheath to perfuse the lower body. A three-way stopcock is placed on the arterial line and connected to the SCP and the arterial sheath.

LBP was established through an ultrasound-guided percutaneous cannulation of the femoral artery with a 20 G, 3 Fr. or 4 Fr. arterial sheath, depending on the size of the common femoral artery and assessment by a congenital cardiac anaesthesiologist. The femoral access was used for two purposes: (a) continuous measurement of intra-arterial pressure and (b) LBP. For the latter, the arterial sheath was connected to the cardiopulmonary bypass (CPB) through a high-flow three-way stopcock and the flow was measured using a flow sensor (Figure 2). During aortic clamping and aortic arch reconstruction, combined SCP and LBP was established and adjusted depending on blood pressure (mean arterial pressure > 40 mmHg) and lactate levels (<3 mmol/L). Patients in this group were cooled down to a target temperature of 30–32 °C. After aortic arch reconstruction was completed, perfusion was established by direct cannulation of the neo-aorta. After surgery and transfer to the paediatric intensive care unit (PICU), the arterial sheath was removed as quickly as possible to avoid any limb malperfusion or ischemia.

### 2.3. Parameter Analysis

Demographic parameters included gender, age, height and weight.

Intraoperative parameters, such as maximum lactate level at reperfusion, duration of cardiopulmonary bypass (CPB), aortic cross-clamping time, lowest temperature, intraoperative packed red blood cells (RBC), fresh frozen plasma (FFP) and thrombocyte pools (TP) were recorded.

Creatinine levels, liver enzymes including alanine aminotransferase (ALAT), aspartate aminotransferase (ASAT), lactate dehydrogenase (LDH) and international standardised ratio (INR) were recorded preoperatively, after transfer to the PICU, 24 h postoperatively and 72 h postoperatively.

At the PICU, examination of the lower extremities was performed on a regular basis, at least once every 8 h, looking for any signs of malperfusion or limb ischemia after sheath removal.

While at the PICU, patients underwent transcranial ultrasound examination for any major congenital malformations before surgery. After surgery, transcranial ultrasound was performed twice a week in all patients; in patients with ECLS, ultrasound was performed daily to check for any bleeding.

Length of stay (LOS) at the PICU, duration of mechanical ventilation (MV) and 30-day mortality were also recorded.

### 2.4. Outcomes

The primary objective was to compare the rate of 30-day mortality. Secondary outcomes were the rates of major early postoperative complications. These included major renal, neurological and gastrointestinal complications. Major renal complications were defined as those requiring renal replacement therapy (RRT). Indications for starting RRT were a GFR < 15 mL/kg/min despite conservative treatment using intravenous diuretics. Major neurological complications included the appearance of neurological symptoms like seizures and intracranial bleeding, or ischemia detected on diagnostic imaging. Major gastrointestinal complications were defined as any impairment of the gastrointestinal system that developed postoperatively and required treatment and prolonging the LOS at the PICU. Multiorgan failure (MOF) was defined as dysfunction of 2 or more organ systems defined by clinical and biochemical parameters.

### 2.5. Statistical Analysis

Statistical analyses were performed using the SSPS 23.0 (IBM Corporation, Armonk, NY, USA) software. Normal distribution was checked using the Kolmogorov–Smirnov test. Continuous variables are reported as means and standard deviation if normally distributed, otherwise as medians and interquartile ranges. Normally distributed variables were compared using Student’s *t*-test and non-normally distributed variables using the Mann–Whitney U test. Ordinal variables are reported as absolute values and percentages and were compared with the Χ^2^ test. Binary regression analysis and a Hosmer–Lemeshow test were performed to calculate regression coefficients and odds ratios.

The ethics commission board approved the study (project number 461/2019BO2). Due to the retrospective nature of the study, written consent was waived.

## 3. Results

There was no significant difference regarding sex, age, length and weight between groups. Preoperative INR values were significantly increased in the WBP group (*p*-value =< 0.01); preoperative renal and hepatic parameters showed no significant difference between groups. Parameters are shown in Table 1.

There was a significantly shorter CPB time (*p*-value =< 0.01) and a trend towards a shorter aortic cross-clamp time in the WBP group (*p*-value = 0.09). The median duration of LBP was 54 ± 39 min. Due to the different temperature goal in each group, a significant difference was expected (*p*-value < 0.01). The highest lactate value recorded during reperfusion was significantly lower in the WBP group (*p*-value < 0.01). The WBP group showed a significantly lower rate of intraoperative RBC, FFP and TP transfusions (*p*-values =< 0.01, <0.01, and <0.01, respectively). Parameters are shown in Table 2.

After arrival at the PICU, patients in the WBP group showed lower ALAT values (*p*-value < 0.01). There was no difference regarding INR, ASAT or LDH values. In the following 24 h, patients in the WBP group showed significantly lower creatinine and higher urine output values (*p*-values = 0.01 and < 0.01), as well as significantly higher INR values (*p*-value = 0.02). After 72 h, a significantly lower creatinine value remained in the WBP group (*p*-value = 0.02), whilst the hepatic, INR and urine output parameters showed no significant difference. Patients in the WBP group had shorter MV times (*p*-value = 0.02), while the LOS at the PICU showed no difference. Parameters are shown in Table 3.

We found no difference regarding the incidence of RRT, NEC or MOF. There was no difference in the incidence of postoperative bleeding complications requiring surgical intervention, the rate of open thorax or need for intra- or postoperative ECLS between groups. We found a significant difference regarding neurological complications in favour of the WBP group (*p*-value = 0.01). There was no difference regarding 30-day mortality. The arterial sheath in the femoral artery was left a median of 1 (1–8) day; four (6.6%) patients suffered from thrombosis of the common femoral artery after sheath removal, but no episodes of malperfusion or limb ischemia were recorded in the entire cohort. Parameters are shown in Table 4.

The binary logistic regression analysis for major postoperative outcomes showed a significant higher risk for RRT, neurological complications, MOF and 30-day mortality in the SCP group. The main predictors for these complications were urine production after 72 h and an intraoperative SCP strategy. Parameters are shown in Table 5.

## 4. Discussion

Our study summarises the results of our 6-year experience of WBP strategy in neonates and infants undergoing aortic arch surgery. A total of 95 patients were included, of which 34 were historical controls in the SCP group and 61 were patients in the WBP group. Our analyses found that patients in the WBP group needed fewer intraoperative transfusions, showed an improvement in postoperative renal function and suffered from fewer neurological complications. Secondary analyses showed that SCP and a deteriorated postoperative renal function may be predictors for RRT, MOF and 30-day mortality. We believe these results should encourage other groups to follow this perfusion strategy in these types of surgeries.

Since the mid-1990s, when the first results in children were published stating that a DCHA for longer than 40 min had long-term neurological consequences for the development of the children, there has been a constant search for the optimal perfusion strategy in neonates and infants [1,2,3]. This led to the development and propagation of the SCP strategy, which, as early as the early 2000s, was shown to provide perfusion of the lower body through collateral vessels [13,14]. In the following years, several groups analysed the effects of SCP and moderate (instead of deep) hypothermia in sensible organs such as the kidneys and the liver [15,16]. Results showed improved renal, hepatic and neurological function following surgery with SCP compared to DHCA [15,16,17,18,19]. Despite the improvement in postoperative outcomes, the search for an even better perfusion was still ongoing.

The first publications to mention an additional LBP to the preexisting SCP date to the early 2010s [6,20]. Several groups have mentioned different strategies to achieve LBP, from cannulating the umbilical artery to the descending aorta and even the femoral artery [6,7,20]. The comparison between SCP and SCP plus LBP was inevitable; the results had shown improved postoperative renal and hepatic function in favour of SCP + LBP [10,11].

Our group was the first to use cannulation of the common femoral artery with an arterial sheath to establish LBP on a routine basis, in what came to be known as WBP [7,11].

The analysis performed showed that there was no difference regarding demographic and preoperative renal and hepatic parameters between groups. The difference seen in the lowest recorded temperatures was expected due to the different temperature goals. The higher temperature goal in the WBP group is possible due to the lack of ischemia of abdominal organs. This absence of ischemia may be supported by the significantly lower maximum lactate value during the reperfusion phase. Interestingly, despite having similar cross-clamp times, the WBP had a significantly shorter CPB time and needed fewer intraoperative RBC, FFP and TP transfusions. We believe this to be the product of having a constant liver perfusion and only mild hypothermia. Improved hepatic function has been described by other groups after using additional LBP [10]. Our group had previously described the need for fewer intraoperative transfusions with a smaller cohort of these patients; we confirmed this with a larger cohort [11].

Upon arrival at the PICU, patients in the WBP had significantly lower ALAT values, which may also be an indicator for an adequate hepatic perfusion; in the first 24 h, this also translated to an improved renal function with significantly lower creatinine and higher urine output values. In the same period, the SCP group showed significantly lower INR values. The reason for this remains uncertain. After 72 h, the WBP group still showed significantly lower creatinine values, despite no significant difference in urine output. These findings also relate to our previous findings and those of other groups [10,12,21,22]. Patients in the WBP group had a significantly shorter duration of mechanical ventilation, which did not translate to a shorter PICU stay. These findings also correlate with the current literature [12,21,22]. The arterial sheath was usually removed within 24 h after surgery; only 6.6% of patients suffered from thrombosis of the common femoral artery after sheath removal. Even though the incidence of this complication is low, it is higher than that reported in cardiologic studies, with an incidence of 1.7% [23]. This higher incidence is probably due to the longer time the sheath stays in place after surgery as opposed to after catheterisation.

Even though WBP showed improved renal and hepatic function during the first 24 h after surgery, this did not translate to significant differences regarding major postoperative complications. There was no difference regarding the rate of open chest after surgery, postoperative bleeding complications, NEC, the need for intra- or postoperative ECLS or MOF. These results speak in favour of the resilience of the renal, hepatic and intestinal systems of neonates to adapt after being subjected to hypothermia and hypoperfusion. These findings concur with the current literature, which report a short-term improvement of the hepatic function in favour of WBP that is not translated to any major complications [10]. Other groups have stated that patients with LBP have a higher postoperative glomerular filtration rate (GFR) or even a significantly lower rate of RRT [8,21]. Interestingly, the WBP group showed a significantly lower rate of neurological complications, mainly intracranial bleeding events, compared to the SCP group (3.3% vs. 17.6%, respectively). In a previous study, we made this same finding with a smaller cohort, where there were no neurological complications in the WBP group [12]. This finding has not been reproduced so far in the literature; other groups have not found any difference between perfusion techniques regarding these types of complications [21,22]. We believe that by achieving a retrograde perfusion through the common femoral artery, collateral vessels aid in the perfusion of the left side of the brain, thus increasing cerebral perfusion pressure and avoiding postoperative bleeding or thrombotic complications.

Although our initial analysis only showed a significant difference in the incidence of neurological complications, it did show a trend with lower incidence of RRT, mortality and MOF in favour of the WBP group. After performing the binary logistic regression analysis, this trend was confirmed, showing statistically significant odds ratios regarding the incidence of RRT, MOF and mortality in WBP patients. A sub-analysis of the predictors leading to these results showed that urine production after 72 h was a predictor for RRT, neurological complications and MOF; the main predictor for 30-day mortality and MOF was having underwent surgery with SCP.

Even though these secondary analyses contain data from a single-centre cohort, we were able to show a statistically significant difference in postoperative renal and hepatic function, which may lead to major postoperative complications or even death.

There was no difference regarding 30-day mortality between groups.

## 5. Limitations

The major limitations of this study are its retrospective nature and the small cohort size. A prospective, multi-centre study would help elucidate the open questions regarding the perfusion types.

## 6. Conclusions

WBP through the femoral artery reduces intraoperative transfusion requirements while improving short-term hepatic and renal function and reducing the incidence of postoperative neurological complications in neonates undergoing complex aortic arch procedures. Our study showed that postoperative renal function and SCP were predictors for RRT, neurological complications, death and MOF.

## Figures and Tables

**Table 1:** Demographic and preoperative parameters.

	Total Cohort N = 95	WBP N = 61	SCP N = 34	*p*-Value
Age (d)	12.5 (8–33.5)	12 (8–45)	13.5 (8–28)	0.7
Sex (m)	63 (66%)	40 (66%)	23 (68%)	0.83
Length (cm)	50 (49–53)	51 (49–54)	50 (49–52)	0.19
Weight (kg)	3.45 (3.1–3.8)	3.5 (3.1–3.9)	3.4 (3.1–3.7)	0.71
Preoperative Parameters				
Creatinine (mg/dL)	0.5 (0.35–0.7)	0.4 (0.3–0.6)	0.55 (0.3–0.7)	0.16
ASAT (mg/dL)	34 (27–48)	34 (27–51)	34 (27–47.5)	0.82
ALAT (mg/dL)	17 (10–30.5)	17 (9.5–29)	17 (11–35)	0.54
LDH (mg/dL)	346.5 (281–452)	349 (284–461)	343.5 (261–442)	0.7
INR	1.2 (1.1–1.2)	1.2(1.1–1.25)	1.1(1–1.2)	<0.01

ALAT: alanine aminotransferase; ASAT: aspartate aminotransferase; INR: international normalised ratio; LDH: lactate dehydrogenase; SCP: selective cerebral perfusion; WBP: whole-body perfusion.

**Table 2:** Intraoperative parameters.

	WBP N = 61	SCP N = 34	*p*-Value
Lowest temperature (°C)	30.86 ± 2.56	26.94 ± 2.13	<0.01
CPB time (min)	133.61 ± 65.97	174.82 ± 64.95	<0.01
Aortic cross-clamp (min)	72.67 ± 44.03	85.72 ± 47.94	0.09
LBP time (min)	54 ± 38.98	N/A	
Max. lactate value during reperfusion (mg/dL)	1.9 (1.6–2.8)	4.3 (3.25–5.55)	<0.01
Intraoperative Transfusions			
RBC (mL)	320 (182.5–560)	600 (300–775)	<0.01
FFP (mL)	280 (150–300)	600 (342.5–600)	<0.01
TP (mL)	60 (25–190)	300 (250–300)	<0.01

CPB: cardiopulmonary bypass; FFP: fresh frozen plasma; LBP: lower-body perfusion; RBC: red blood cells; TP: thrombocyte pool; SCP: selective cerebral perfusion; WBP: whole-body perfusion.

**Table 3:** Postoperative parameters.

	WBP N = 61	SCP N = 34	*p*-Value
Preoperative Parameters			
ALAT (mg/dL)	72 (48.5–104)	84 (62–135)	0.1
ASAT (mg/dL)	13 (9–19.5)	20.5 (16–28)	<0.01
LDH (mg/dL)	408 (346.5–553.5)	445.5 (349–543)	0.4
INR	1.1 (1–1.2)	1.1 (1–1.1)	0.1
Postoperative Parameters after 24 h			
Creatinine (mg/dL)	0.52 ± 0.21	0.64 ± 0.25	0.02
ALAT (mg/dL)	51 (37–100.5)	49 (37–152)	0.78
ASAT (mg/dL)	10 (8–16.5)	12.5 (8–25)	0.23
LDH (mg/dL)	361 (287.5–530)	374.5 (315–669)	0.3
INR	1.2 (1.1–1.3)	1.1 (1.1–1.2)	0.02
Urine output (mL/h)	6.54 (4.63–10.17)	3.46 (2.88–4.71)	<0.01
Postoperative Parameters after 72 h			
Creatinine (mg/dL)	0.5 (0.4–0.6)	0.6 (0.5–0.9)	0.02
ALAT (md/dL)	30.5 (20–46.5)	24 (17–45)	0.5
ASAT (md/dL)	8.5 (5–15)	7.5 (4–14)	0.33
LDH (md/dL)	326 (261–539)	326.5 (256–485)	0.98
INR	1.1 (1.05–1.2)	1.1 (1–1.2)	0.41
Urine output (mL/h)	23.25 (19.25–27.17)	20.77 (14.38–26.50)	0.06
Duration of mechanical ventilation (d)	6 (3–11)	8 (6–14)	0.02
Length of stay at the PICU (d)	13 (8–26)	15 (9–28)	0.38

ALAT: alanine aminotransferase; ASAT: aspartate aminotransferase; INR: international normalised ratio; LDH: lactate dehydrogenase; PICU: paediatric intensive care unit; SCP: selective cerebral perfusion; WBP: whole-body perfusion.

**Table 4:** Postoperative complications.

	WBP N = 61	SCP N = 34	*p*-Value
RRT	4 (6.6%)	5 (14.7%)	0.19
Neurological deficits	2 (3.3%)	6 (17.6%)	0.01
Open thorax	34 (55.7%)	22 (64.7%)	0.39
NEC	8 (13.1%)	4 (11.8%)	0.85
Postoperative bleeding complications	8 (13.1%)	5 (14.7%)	0.82
Intraoperative ECLS	14 (23.0%)	8 (23.5%)	0.95
Postoperative ECLS	7 (11.5%)	1 (2.9%)	0.15
Punction-related complications	4 (6.6%)	-	
Multiorgan failure	1 (1.6%)	2 (5.9%)	0.25
30-day mortality	3 (4.9%)	2 (5.9%)	0.84

ECLS: extracorporeal life support; NEC: necrotising enterocolitis; RRT: renal replacement therapy; SCP: selective cerebral perfusion; WBP: whole-body perfusion.

**Table 5:** Binary logistic regression and odds ratios of major postoperative complications between whole-body perfusion vs. selective cerebral perfusion.

Outcome	Regression Coefficient	Odds Ratio	CI (95%)	*p*-Value
Dialysis	−0.011	0.989	0.983–0.995	<0.01
Neurological deficits	−0.006	0.994	0.988–1	0.06
30-day mortality	−3.346	0.035	0.003–0.391	<0.01
NEC	0.018	1.018	1.001–1.035	0.04
Multiorgan failure	−6.003	0.002	0–0.275	0.01

NEC: necrotising enterocolitis.

## Data Availability

The data may be available upon request.

## Abbreviations

CPB: cardiopulmonary bypass; DHCA: deep hypothermic circulatory arrest; ECLS: extracorporeal life support; FFP: fresh-frozen plasma; LBP: lower-body perfusion; LOS: length of stay; MOF: multiorgan failure; MV: mechanical ventilation; NEC: necrotising enterocolitis; PICU: paediatric intensive care unit; RBC: red blood cells; RRT: renal replacement therapy; SCP: selective cerebral perfusion; TP: thrombocyte pool; WBP: whole-body perfusion.
